# Matrix Metalloproteinase-9 Expression Is Enhanced in Renal Parietal Epithelial Cells of Zucker Diabetic Fatty Rats and Is Induced by Albumin in *In Vitro* Primary Parietal Cell Culture

**DOI:** 10.1371/journal.pone.0123276

**Published:** 2015-04-07

**Authors:** Yuanyuan Zhang, Jasmine George, Yun Li, Rebecca Olufade, Xueying Zhao

**Affiliations:** Department of Physiology, Morehouse School of Medicine, Atlanta, Georgia, 30310, United States of America; Fondazione IRCCS Ospedale Maggiore Policlinico & Fondazione D’Amico per la Ricerca sulle Malattie Renali, ITALY

## Abstract

As a subfamily of matrix metalloproteinases (MMPs), gelatinases including MMP-2 and MMP-9 play an important role in remodeling and homeostasis of the extracellular matrix. However, conflicting results have been reported regarding their expression level and activity in the diabetic kidney. This study investigated whether and how MMP-9 expression and activity were changed in glomerular epithelial cells upon albumin overload. In situ zymography, immunostaining and Western blot for renal MMP gelatinolytic activity and MMP-9 protein expression were performed in Zucker lean and Zucker diabetic rats. Confocal microscopy revealed a focal increase in gelatinase activity and MMP-9 protein in the glomeruli of diabetic rats. Increased glomerular MMP-9 staining was mainly observed in hyperplastic parietal epithelial cells (PECs) expressing claudin-1 in the diabetic kidneys. Interestingly, increased parietal MMP-9 was often accompanied by decreased staining for podocyte markers (nephrin and podocalyxin) in the sclerotic area of affected glomeruli in diabetic rats. Additionally, urinary excretion of podocyte marker proteins was significantly increased in association with the levels of MMP-9 and albumin in the urine of diabetic animals. To evaluate the direct effect of albumin on expression and activity of MMP-9, primary cultured rat glomerular PECs were incubated with rat serum albumin (0.25 - 1 mg/ml) for 24 - 48 hrs. MMP-9 mRNA levels were significantly increased following albumin treatment. Meanwhile, albumin administration resulted in a dose-dependent increase in MMP-9 protein and activity in culture supernatants of PECs. Moreover, albumin activated p44/42 mitogen-activated protein kinase (MAPK) in PECs. Inhibition of p44/42 MAPK suppressed albumin-induced MMP-9 secretion from glomerular PECs. Taken together, we have demonstrated that an up-regulation of MMP-9 in activated parietal epithelium is associated with a loss of adjacent podocytes in progressive diabetic nephropathy. Albumin overload may induce MMP-9 expression and secretion by PECs via the activation of p44/42 MAPK pathway.

## Introduction

Diabetic nephropathy is now the leading cause of end-stage renal disease, accounting for 40–50% of the patients entering dialysis each year in the United States. Albuminuria not only serves as a marker for early kidney injury, but also plays a central role in the pathogenesis of progressive renal dysfunction. In the past decades, studies have highlighted the importance of podocytes, the terminally differentiated visceral epithelial cells of the glomerulus, in the pathogenesis of proteinuric diseases. It is clear that primary podocyte injury can induce the development of proteinuria and glomerulosclerosis [1–3]. However, in most studies, a reduced number of podocytes is associated with the presence of a glomerular scar, even if the initial injury was not directed primarily against podocytes [2]. It has been suggested that podocytes can also be lost secondarily because of the invasion of parietal epithelial cells (PECs) in glomerular diseases [2].

Glomerular parietal epithelial cell, the second glomerular epithelial cell type, has recently gained increasing interest because of current understanding of how PEC biology underlies normal function in health and how in glomerular diseases PECs may serve a critical reparative role, or under different circumstances the response by PECs may lead to further glomerular damage [4–7]. The PECs of Bowman’s capsule not only play a role as a second glomerular barrier for limiting filtered albumin from exiting the urinary space [4] but may also have the capacity to differentiate into podocytes during diseases [5]. Moreover, a close relationship between the PECs and podocytes has been suggested by the findings that PEC activation is accompanied by progressive podocytopenia in cellular/collapsing focal segmental glomerulosclerosis (FSGS) [2, 8] and diabetic glomerulosclerosis [9]. Nevertheless, it is still unclear what stimulates PEC migration into the capillary tuft and promotes podocyte injury in proteinuric kidney disease.

Matrix metalloproteinases (MMPs) are a group of zinc-dependent enzymes with proteolytic activity against extracellular matrix (ECM) proteins. MMPs were previously known to be anti-fibrotic for their ability to degrade and remodel ECM. Recent studies have shown that MMPs are implicated in initiation and progression of kidney fibrosis [10]. For example, increased expression of MMP-2 and MMP-9 has been shown to be associated with the induction of tubular cell epithelial-mesenchymal transition (EMT) in vitro [11] and in vivo [12]. A decrease in MMP-9 induction has been suggested to be responsible for the beneficial outcome of tPA deficiency via the preservation of tubular basement membrane and avoidance of tubular EMT as seen in wild-type kidneys [12]. The role of MMPs in the pathogenesis of diabetic glomerulopathy appears to be complex. In fact, conflicting results have been reported regarding renal expression and activity of MMP gelatinases in diabetic nephropathy. For example, studies performed in streptozotocin-induced diabetic rats indicated that decreased expression and activity of MMP-2 and MMP-9 contribute to mesangial matrix accumulation in the kidney [13–14]. In contrast, there are increasing evidences that glomerular MMP-9 protein expression and catalytic activity are enhanced in diabetic nephropathy and that the suppression of renal MMP-9 expression by genetic defect [15] or pharmacological interventions [16–17] attenuates diabetic nephropathy.

Here, using in situ zymography and immunostaining we investigated the pattern and cellular origin of glomerular MMP-9 expression and activity in Zucker diabetic fatty rat, an animal model of type 2 diabetes mellitus. In addition, we also attempted to evaluate the direct effect of albumin overload (mimicking albuminuria) on MMP-9 expression and activity in primary cultured glomerular PECs.

## Materials and Methods

### Animals

Male Zucker lean and Zucker diabetic fatty rats were purchased from Charles River Laboratories (Wilmington, MA). Rats were housed in a temperature-controlled room with a 12:12-h light-dark cycle and free access to Purina 5008 rat chow and water. Urine was collected over a 24-h period in metabolic cages and stored at -80°C until use. Blood glucose was monitored using the Accu-chek glucometer by tail-vein blood sampling. This study was carried out in strict accordance with the recommendations in the Guide of the Care and Use of Laboratory Animals of the National Institutes of Health. All animal protocols were approved by the Institutional Animal Care and Use Committee of the Morehouse School of Medicine (approval number 12–27). All surgery was performed under sodium pentobarbital anesthesia, and all efforts were made to minimize suffering.

### Histology and in situ Zymography

Kidney tissue was collected from Zucker lean and Zucker diabetic fatty rats at 20 and 28 weeks of age. Formalin-fixed, paraffin-embedded kidney tissue was sectioned and stained with hematoxylin and eosin (HE), and Masson’s trichrome stains. The gelatinolytic activity of MMPs was examined in 5-μm-thick cryostat sections of OCT-embedded kidney tissues using in situ zymography with Fluorescein conjugated, dye-quenched gelatin from pig skin (DQ-gelatin, Life Technologies, Grand Island, NY) as previously described [18]. Briefly, one milligram DQ gelatin was dissolved in 1 ml Milli-Q water and further diluted 1:50 in a reaction buffer containing 50 mM Tris-HCl, 150 mM NaCl, and 5 mM CaCl_2_ (pH 7.6). This substrate for gelatinases was dropped on tissue sections, covered with Parafilm, and incubated in a dark humidity chamber at 37°C for 2 hrs. Then the Parafilm was gently removed. The sections were washed with PBS and fixed in 4% buffered paraformaldehyde solution for 10 mins in the dark. The slides were washed again and mounted with Fluoromount. The level of autofluorescence in the tissue was evaluated by substrate incubation on control sections from each tissue at -20°C. The sections were observed and imaged by Leica confocal fluorescence microscope. Proteolytic activity was detected as bright green fluorescence, which indicates substrate breakdown.

### Immunofluorescence Staining

To study the relationship between gelatinolytic activity and MMP-9 expression, 5-μm-thick cryostat sections were first incubated with DQ gelatin as described above, and then stained for MMP-9. To further examine the cellular origin of glomerular MMP-9, dual labeling was performed by incubating kidney sections with a mixture of two antibodies overnight: rabbit anti-MMP-9 (abcam, Cambridge, MA) with goat anti-podocalyxin (R&D Systems, Carlsbad, CA), guinea pig anti-nephrin (Fitzgerald, Concord, MA), rabbit anti-Wilms tumor (WT)-1 (Santa Cruz Biotechnology, Santa Cruz, CA), mouse anti-desmin (Dako, Carpinteria, CA), or mouse anti-claudin-1 (Life Technologies). The number of podocytes was identified by nuclear WT-1 staining per 5-μm kidney section, and WT-1 positive cells were counted in at least 50 glomeruli per animal. As a negative control, the sections were exposed to nonimmune IgG (in replacement of primary antibodies) with the same secondary antibodies, and no specific staining occurred. The sections were observed and imaged by Leica confocal microscope.

### Culture of Primary Parietal Epithelial Cells

Glomeruli were isolated from Sprague-Dawley rat kidneys by a modified procedure as described previously [19]. The glomerular tissue fragments were collected and suspended in DMEM/F12 medium (Life Technologies), supplemented with 5% fetal bovine serum, penicillin and streptomycin. To test the effect of albumin on MMP-9 expression and activity, 80–90% confluent glomerular PECs (day 6) were washed with serum-free DMEM and incubated in DMEM/F12 with rat serum albumin (RSA, Sigma Aldrich Inc., St. Louis, MO) at varying concentrations (0, 0.25, 0.5, or 1 mg/ml) for an additional 24 or 48 hrs. This preparation of RSA has been shown to be essentially fatty acid free and very low endotoxin by the manufacturing company and the range of albumin concentrations is similar to that used in the previous study [20]. In another set of experiment, the cells were treated with 0.25 mg/ml RSA for 1.5, 3 or 6 hrs. Following the treatment, the cells were collected and the cell pellets were lysed using PhosphoSafe Extraction Reagent (MED Millipore, Temecula, CA) containing a cocktail of protease inhibitors (Sigma Aldrich Inc.). To evaluate the role of p44/42 MAP kinase in albumin-mediated MMP-9 induction, the cells were pretreated with U0126 [1,4-diamino-2,3-dicyano-1,4-bis (2-amino phenylthio) butadiene, Sigma Aldrich Inc.], a selective p44/42 inhibitor. Culture supernatants were collected, and detached cells were removed by centrifugation. The samples were stored at -80°C for Western blot and gelatin zymography analyses.

To evaluate the effect of high glucose on MMP-9 production, PECs were incubated with DMEM/F12 containing either 5 or 30 mM D-glucose medium for 24 or 48 hrs. The effect of hyperosmolality was assessed in PECs cultured in DMEM/F-12 containing 5 mM D-glucose supplemented with 25 mM mannitol. In another set of experiment, the PECs were cultured in DMEM/F12 medium for 24–48 hrs in the absence or presence of 1 or 2 ng/ml TGF-β1 (R&D Systems). The samples were collected and processed as above.

### Taqman and quantitative real-time PCR analysis

Total RNA was prepared from primary PECs by using ultra-pure TRIzol reagent according to the manufacturer's instructions (GIBCO-BRL, Grand Island, NY). MMP-2, MMP-9 and β-actin gene-specific Taqman probe and primer sets were obtained from Applied Biosystems (Applied Biosystems Ins., Foster City, CA) as Assays-on-Demand gene expression products. The Assays-on-Demand identification numbers were Rn02532334_s1 for MMP-2, Rn01423075_g1 for MMP-9, and 4331182 for rat β-actin endogenous control. Each sample was run in triplicate, and the comparative threshold cycle (C_t_) method was used to quantify fold increase (2^–ΔΔCt^) compared with normal controls.

### Gelatin Zymography

The gelatinolytic activity of MMP was examined in rat urine samples and culture supernatants from primary PECs as described previously [21–22]. Briefly, samples were resolved by electrophoresis in a 10% polyacrylamide gel containing gelatin (1mg/ml). After running, the gels were washed four times in renaturing buffer (2.5% Triton X-100) for 15 mins each before incubating for 16–24 hrs at 37°C in developing buffer. The gels were stained with Coomassie Brilliant Blue R-250 (Sigma Aldrich Inc.) for 30 mins. After washing with a destaining solution [Methanol: Acetic acid: Water (50: 10: 40)], the gelatinolytic activity was visualized as a clear band in the uniformly stained background. The gels were scanned using white light transillumination, and the relative density of each gelatinolytic band was determined using ImageJ software.

### Immunoblot analysis

Rat urine samples, culture supernatants or cell lysates were separated by 10% SDS-PAGE and transferred electrophoretically to nitrocellulose membrane. The blots were incubated with antibodies for MMP-9, nephrin, synaptopodin (Santa Cruz Biotechnology Inc., Santa Cruz, CA), or claudin-1. Some membranes were first hybridized with phospho-specific p44/42 or p38 MAPK antibodies (Cell signaling Technology, Danvers, MA), stripped, and then reprobed with an antibody that recognizes total p44/42 MAPK or total p38 MAPK (Cell signaling). Detection was accomplished by enhanced chemiluminescence Western blotting (ECL, GE Healthcare, Piscataway, NJ). Relative band intensity was measured densitometrically.

### Statistical analysis

Data are expressed as mean±SEM. Student’s *t* test was used for comparison between two groups. Comparisons among multiple groups were performed by one-way ANOVA followed by Newman-Keuls post hoc test. Statistical significance was set at *P*<0.05.

## Results

### Glomerular Gelatinolytic Activity Detected by in Situ Zymography in Relation to Localization of MMP-9 in Rat Kidney Tissues

**Fig 1:**
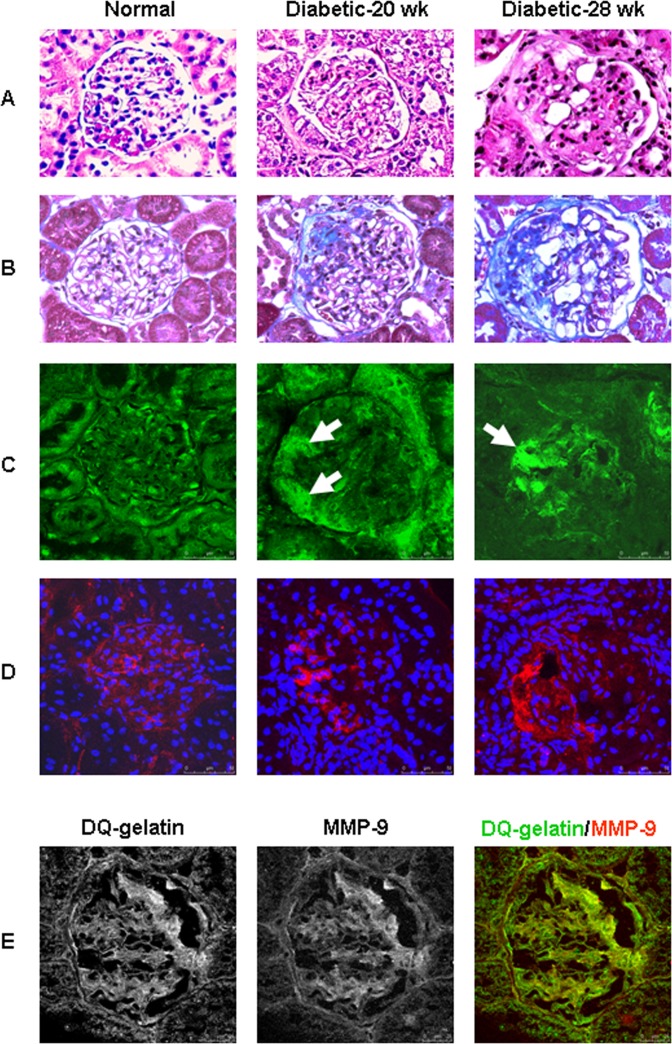
Focally increased gelatinolytic activity and MMP-9 protein in the glomeruli of Zucker diabetic fatty rats. A and B: Hematoxylin-eosin (A) and Masson’s trichrome (B) stains of paraffin kidney sections show normal glomeruli of Zucker lean (Normal), and progressive glomerulosclerosis in 20 to 28-week-old Zucker diabetic fatty rats (Diabetic-20 wk and 28 wk). C: In situ zymography shows gelatinolytic activity (bright green) in individual glomerular cells of normal rats. In diabetic rats, focally increased gelatinolytic activity was observed at the periphery of glomerulus (Diabetic-20 wk, white arrow) and in continuity onto the capillary tuft (Diabetic-28 wk, white arrow). D: Individual MMP-9 positive cells (red) are shown throughout the glomerular tuft in a normal glomerulus. Enhanced MMP-9 signal is seen in the sclerotic lesions in glomeruli of diabetic rats. E: Dual labeling for FITC-conjugated DQ-gelatin (green) and MMP-9 (red) shows a colocalization of gelatinolytic activity and MMP-9 protein in rat glomeruli.

Dysregulation of MMPs expression and activity has been reported in various glomerular diseases including diabetic nephropathy. Using gelatin in situ zymography, we were able to assess net proteolytic activity of MMP gelatinases in the glomeruli of normal and diabetic kidneys. In normal rat kidneys, gelatinolytic activity was identified in individual cells throughout the entire glomerular tuft (Fig 1C, Normal). In contrast, focally increased gelatinolytic activity was recognized at the periphery of the glomeruli in 20-week-old Zucker diabetic rats (Fig 1C, Diabetic-20 wk). The enhanced local proteolytic activity was frequently associated with morphologic changes in the activated cells [i.e., increased cellular volume and numbers, thickening of the basement membrane of Bowman’s capsule (BC), and focal fibrotic lesion], as shown by HE and Masson’s trichrome stains (Fig 1A and 1B, Diabetic-20 wk). In more advanced stages, extensive fibrotic/sclerotic lesions were often seen in affected glomeruli (Fig 1A and 1B, Diabetic-28 wk). Increased gelatinolytic activity was present within the sclerotic lesion and covered large segments of the glomerular tuft (Fig 1C, Diabetic-28 wk). Gelatinase activity was also enhanced in the tubules of diabetic rats.

We next performed immunofluorescence staining to examine the expression pattern of MMP-9 in the glomeruli of Zucker rats. Similar to the distribution pattern of gelatinolytic activity, individual cells with strong MMP-9 signal were mainly located in the mesangium with weak but continuous linear staining along BC (Fig 1D, Normal) in normal glomeruli. Diabetes was associated with a focal increase in MMP-9 protein in the glomeruli. Initially, increased MMP-9 positive cells were often observed at the periphery of the glomerulus in the diabetic rats (Fig 1D, Diabetic-20 wk). With disease progression, enhanced MMP-9 signal was also present on the glomerular tuft (Fig 1D, Diabetic-28 wk). Dual labeling for DQ-gelatin and MMP-9 revealed that glomerular gelatinolytic activity was colocalized with MMP-9 protein in rat kidneys (Fig 1E).

### Parietal MMP-9 Induction in Association with a Decrease in Normal Podocyte Markers in the Glomeruli of Diabetic Rats

**Fig 2:**
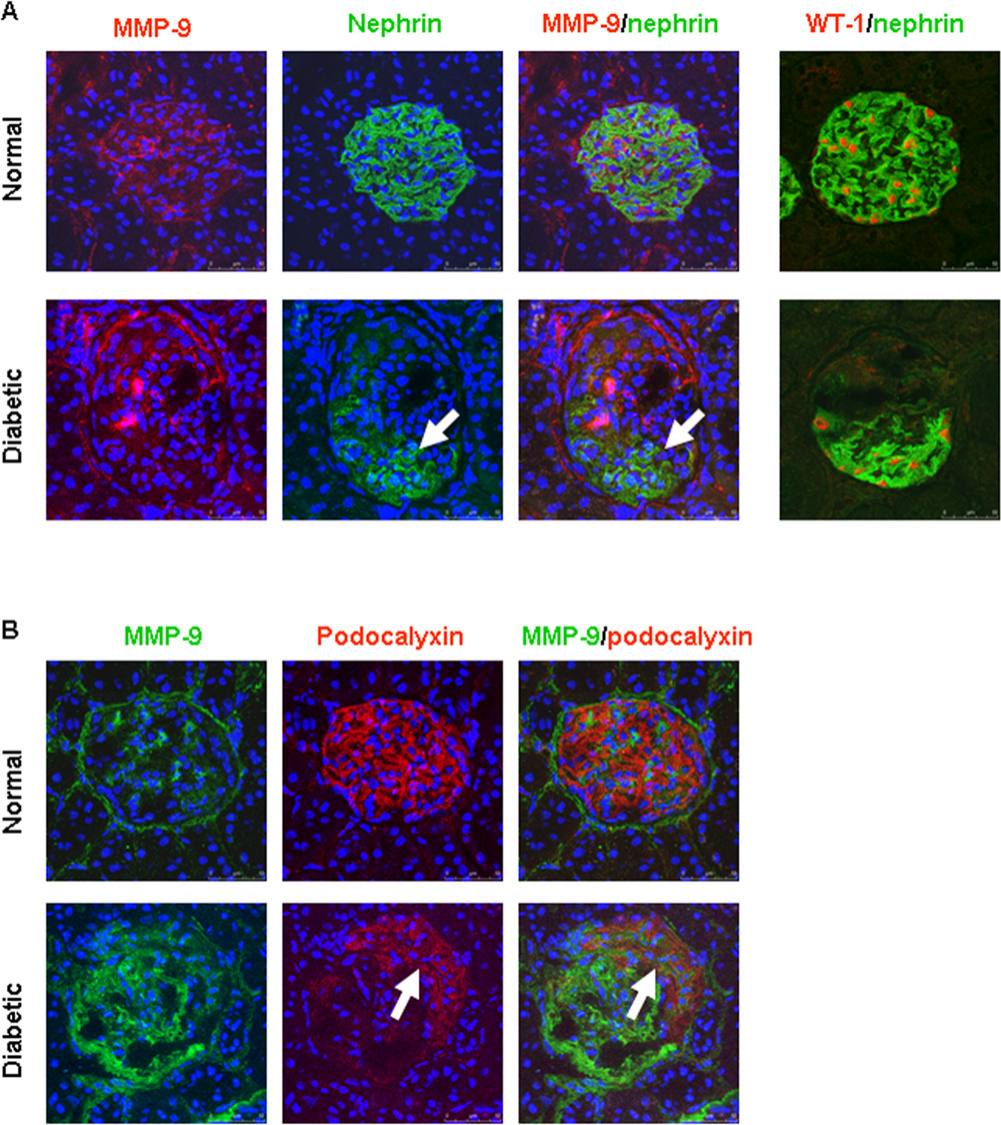
Representative confocal images of dual labeling for MMP-9 and podocyte marker protein in the glomeruli of Zucker rats. Normal glomerulus presents nephrin (A-normal: green) and podocalyxin (B-normal: red) positive staining covering the entire capillary tuft. In the glomeruli of diabetic kidneys, neither nephrin (A-diabetic) nor podocalyxin (B-diabetic) was expressed in sclerotic lesions, where was often populated by MMP-9 positive cells. The podocyte markers, nephrin and podocalyxin, are preserved along the remaining intact portion of the glomerular tuft (white arrows). Dual labeling for nephrin and WT-1 shows that WT-1 positive cells are within nephrin-positive area in the glomerular tuft (A). A significant decrease in the number of WT-1 positive cells was observed in the diabetic glomeruli (A-Diabetic).

An increase in podocyte MMP-9 production has been demonstrated in the diabetic mouse kidneys [15]. In the current study, we carried out multiple labeling for MMP-9 with nephrin or podocalyxin to further characterize the intraglomerular expression of MMP-9 in rat kidney tissue. As depicted in Fig 2, the podocyte markers nephrin and podocalyxin were expressed throughout the entire capillary tuft in normal glomeruli. There was no significant colocalization between MMP-9-positive cells and podocytes. Diabetes was associated with a decrease in podocyte marker proteins. In the diabetic glomeruli with sclerotic lesions, enhanced MMP-9 staining was always observed in association with the affected sclerotic segment, whereas the expression of nephrin or podocalyxin was excluded from this area (Fig 2, diabetic). Nephrin and podocalyxin expression was preserved in the remaining unaffected part of the glomerular tuft (Fig 2, diabetic). In normal control rats, the number of WT-1 positive podocytes (13.8 ± 0.3 cells/glomerular cross-section, n = 4) was similar to those reported by other groups [23]. The number of podocytes was significantly decreased in the glomeruli of 20-week-old Zucker diabetic rats (9.0 ± 0.8 cells/glomerular cross-section, n = 5, *P* < 0.05).

**Fig 3:**
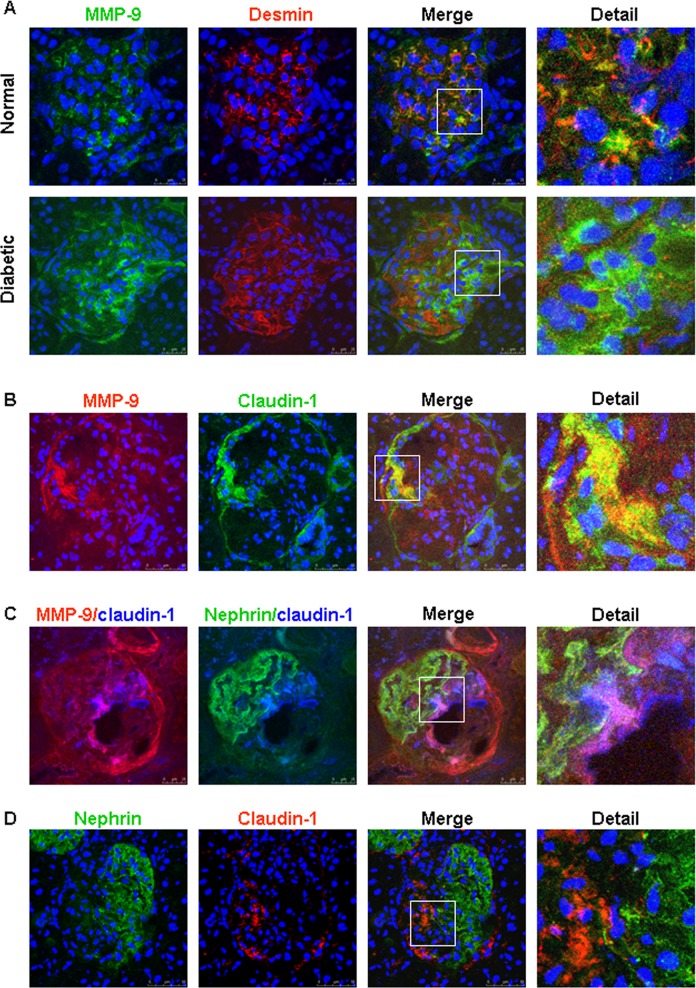
Representative confocal images of multiple labeling for MMP-9 and mesangial or parietal epithelial cell marker proteins in the glomeruli of Zucker rats. A: In normal rat kidney tissue, MMP-9 (green) is mainly present in desmin (red)-positive mesangial cells (A-Normal). In the diabetic glomeruli, increased MMP-9 green signal was often observed in the area with low desmin staining (A-Diabetic). B: In the diabetic rats, enhanced MMP-9 (red) signal is present in parietal epithelial cells expressing claudin-1. C: Triple staining for MMP-9 (red), claudin-1 (blue) and nephrin (green) in a glomerulus of diabetic rats. D: Dual labeling for nephrin (green) and claudin-1 (red) in the diabetic kidney.

Since MMP-9 expression has been observed in mesangial cells [24], we further performed dual labeling for MMP-9 and desmin, a marker for mesangial cells [4, 25]. As shown in Fig 3A-Normal, a colocalization of MMP-9 and desmin was detected in the mesangial area of normal rat kidneys. In contrast, we often detected increased MMP-9 signal in the area with low desmin staining in damaged glomeruli of diabetic rats (Fig 3A-Diabetic). In the diabetic kidneys, double staining for MMP-9 and claudin-1 further revealed that strong MMP-9 staining was largely within claudin-1-positive parietal epithelium (Fig 3B). Moreover, increased MMP-9 and claudin-1 positive cells were often present in area with extensive loss of nephrin expression in the diabetic glomeruli (Fig 3C and 3D).

### Urinary Excretion of MMP-9 and Podocyte Marker Proteins by Zucker Diabetic Rats

**Fig 4:**
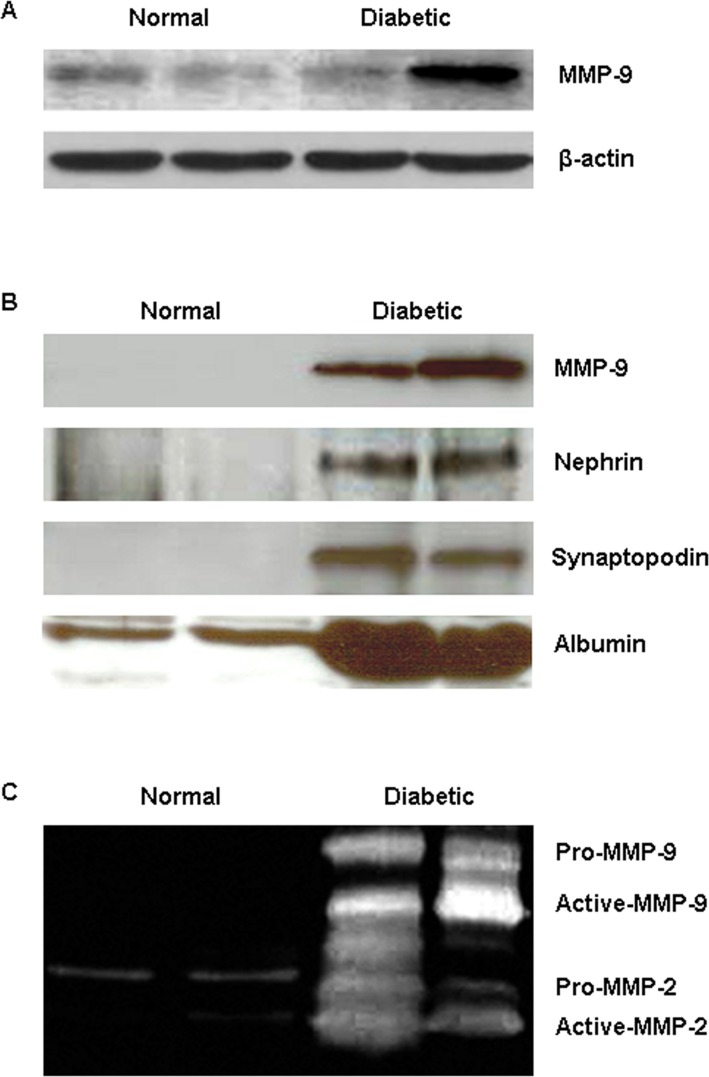
Increased urinary excretion of MMP-9 and podocyte marker proteins in Zucker diabetic rats. (A) Western blot analysis of renal cortical MMP-9 protein in 20-week-old Zucker lean controls and diabetic rats. (B) Representative Western blot images show a significant increase in urinary MMP-9 and podocyte marker proteins, nephrin and synaptopodin, in association with albuminuria in 20-week-old Zucker diabetic rats compared to normal controls. (C) Gelatin zymography analysis confirms a massive increase in urinary MMP-9 and MMP-2 activities in the diabetic rats.

To quantify protein level of MMP-9 in kidney tissue, Western blot analysis was performed with whole cell homogenates of renal cortex isolated from 20-week-old Zucker lean and diabetic rats. As shown in Fig 4A, renal cortical MMP-9 protein tends to increase in the diabetic rats (0.473±0.175, n = 4) compared to age-matched controls (0.249±0.043, n = 4), but this difference did not reach statistical significance (*P* = 0.13). Next, we examined urinary MMP-9 protein and activity in correlation with the excretion of podocyte marker proteins in the diabetic rats. Western blot analysis revealed a massive increase in urinary MMP-9 protein in 20-week-old diabetic rats, which was associated with an increase in urinary excretion of podocyte marker proteins, nephrin and synaptopodin (Fig 4B). The increased excretion of MMP-9 and podocyte marker proteins was positively correlated with urinary albumin level in the diabetic rats. Gelatin zymography further confirmed an elevation of gelatinase activity in the urine of diabetic animals. As shown in Fig 4C, urinary MMP-9 (both pro- and active-) was dramatically increased in 20-week-old diabetic rats, whereas it is undetected in normal urine. Increased MMP-2 activity was also observed in the urine of diabetic rats.

### Effect of Albumin on MMP-9 Expression and Secretion in Primary Glomerular PECs

**Fig 5:**
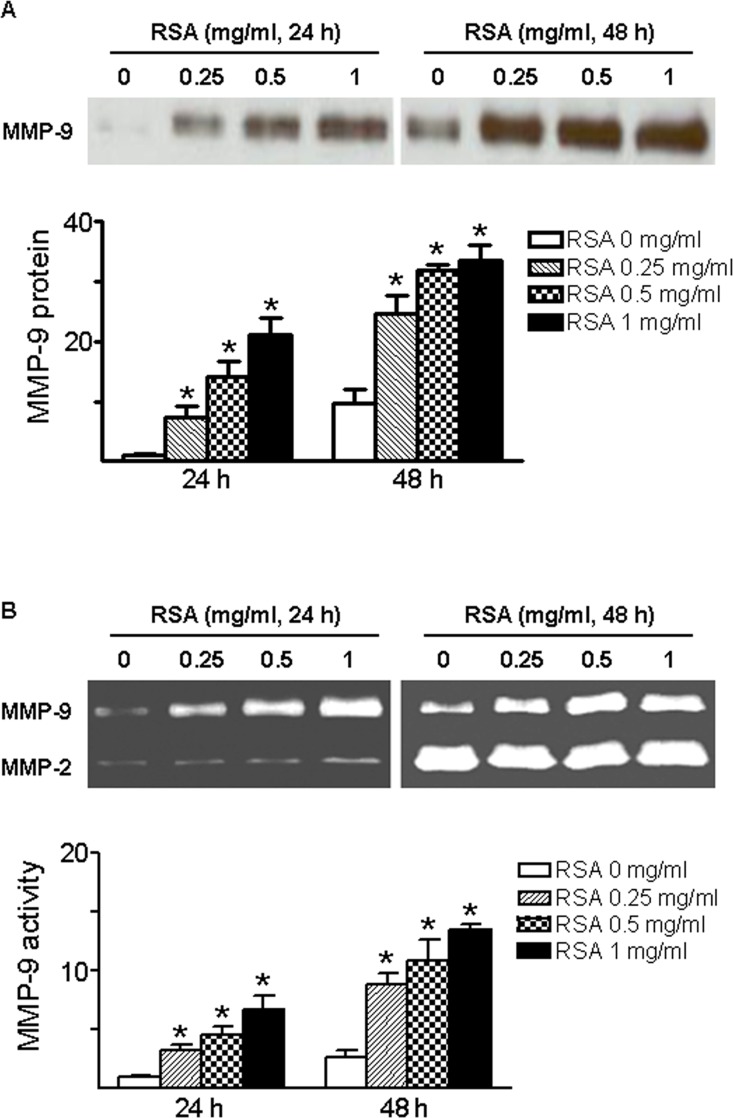
Effect of albumin overload on MMP-9 production by primary glomerular PECs. Rat serum albumin (RSA 0.25–1 mg/ml) administration resulted in a dose-dependent increase in MMP-9 protein (A) and activity (B) in culture supernatants of primary rat glomerular PECs. Values are mean±SEM. An n of 4–6 epithelial cultures were treated for each condition; *P<0.05 vs. untreated control group.

**Fig 6:**
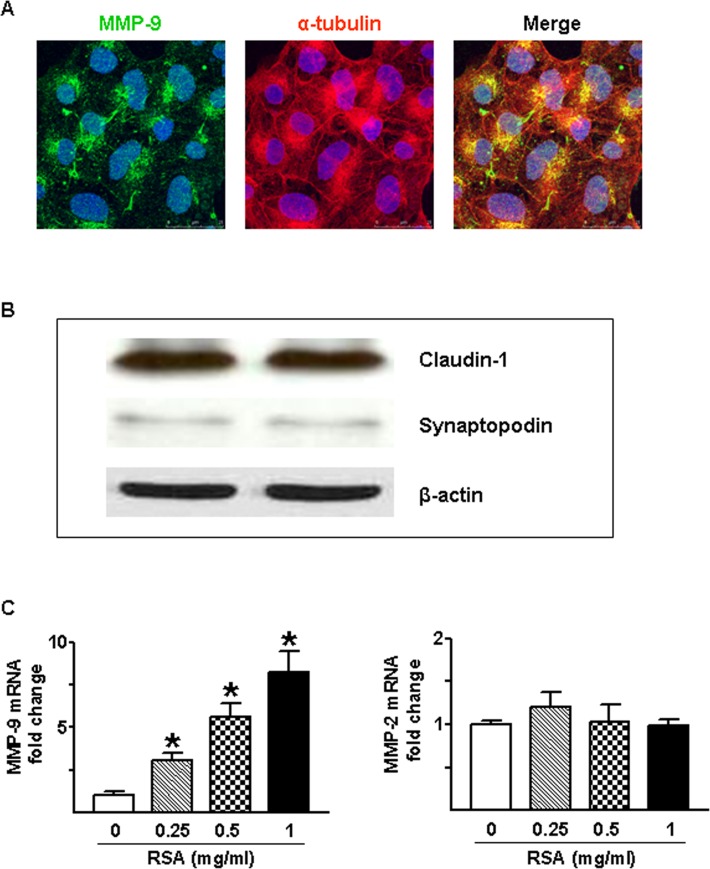
Expression and localization of MMP-9 in glomerular PECs. Dual labeling using antibodies specific for MMP-9 (green) and α–tubulin (red) reveals a vesicular staining pattern that MMP-9 localizes on most microtubules (A) in rat glomerular PECs. Western blot analysis shows that primary PECs express high-level of claudin-1 and low-level of synaptopodin (B). Taqman real-time PCR analysis shows a dose-dependent increase in MMP-9 mRNA when the PECs were incubated with rat serum albumin (RSA, 0.25–1 mg/ml) for 24 hrs (C), whereas MMP-2 mRNA level was not affected. Values are mean±SEM. An n of 4–6 epithelial cultures were treated for each condition; *P<0.05 vs. untreated control group.

Next, we directly evaluated the effect of albumin on MMP-9 expression and secretion using primary cultured rat glomerular PECs. The PECs were incubated with different concentrations of rat serum albumin (RSA, 0.25–1 mg/ml) for 24 or 48 hrs. MMP-9 protein in control supernatants was low at 24 hr and moderately increased at 48 hr (Fig 5A). Albumin administration resulted in dose-dependent increase in MMP-9 protein in the culture supernatants of PECs. This is confirmed by the zymography results that MMP-9 activity was increased in a dose-dependent manner when the PECs were exposed to RSA for 24 or 48 hrs (Fig 5B). In contrast to the stimulatory effect of albumin on MMP-9, MMP-2-related gelatinase activity was constantly detected in control media especially at 48 hr, and not significantly affected by albumin treatment. Immunofluorescence labeling further visualized the intracellular localization of MMP-9 protein in primary cultured PECs. As shown in Fig 6A, MMP-9 was primary present in small cytoplasmic vesicles that are linked to microtubules in glomerular PECs. As depicted in Fig 6B, Western blot analysis revealed that claudin-1 was predominantly expressed in primary cultured glomerular cells, whereas synaptopodin protein level was relatively low in cellular homogenates.

To further investigate whether sustained increase in the lytic content of MMP-9 by RSA is due to an increase in the expression level of MMP-9 gene, we performed real-time PCR analysis. Concomitantly with the stimulatory effect on gelatinolytic MMP-9 content, RSA dose-dependently increased MMP-9 mRNA level (Fig 6C), but had no effect on MMP-2 expression in primary PECs. These data indicate that albumin-induced extracellular MMP-9 content predominantly results from increased MMP-9 gene expression.

### Signaling Mechanism of MMP-9 Upregulation upon Albumin Stimulation

**Fig 7:**
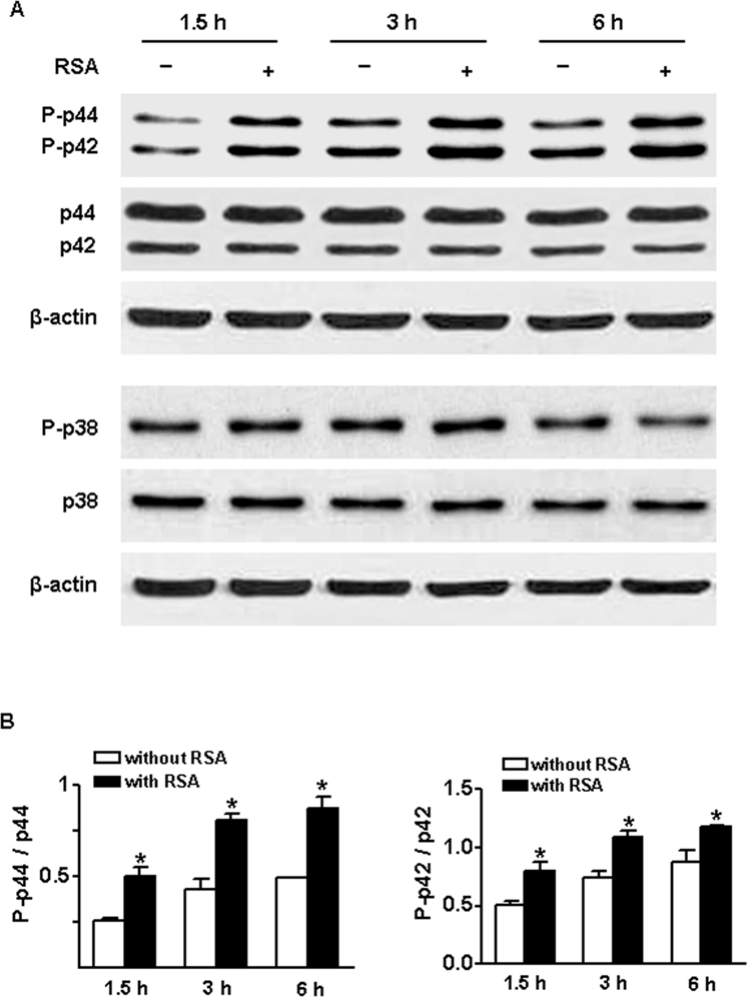
Albumin activates p44/42 MAP kinase in glomerular PECs. (A) Representative Western blots show protein levels of phospho- and total p44/42 and p38 MAP kinases in PECs in the absence or presence of rat serum albumin (RSA, 0.25 mg/ml) for 1.5, 3 or 6 hrs. (B) Quantitative data show activation of p44/42 MAPK in response to albumin stimulation. Values are mean±SEM. An n of 4 epithelial cultures were treated for each condition; *P<0.05 vs. untreated normal control.

**Fig 8:**
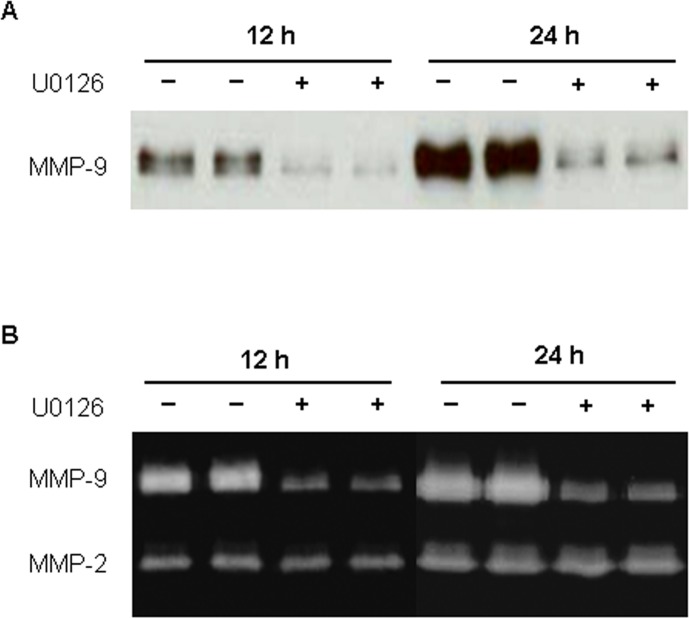
Inhibition of p44/42 MAPK blocks albumin-induced MMP-9 in glomerular PECs. Western blot (A) and gelatin zymography (B) analyses show that pre-treatment of PECs with U0126, a selective p44/42 MAPK inhibitor, significantly reduced MMP-9 protein and activity in culture supernatants of PECs treated with rat serum albumin (0.25 mg/ml) for 12 and 24 hrs.

MMP-9 expression and secretion have been shown to be mediated through activation of the MAPK and PI3K/Akt signaling pathways in various cell types with a variety of stimuli [26–27]. These findings led us to ask whether activation of MAPK and PI3K/Akt pathways is also involved in albumin-induced MMP-9 production by glomerular PECs. Thus, the PECs were lysed at 1.5, 3 and 6 hr following exposure to 0.25 mg/ml RSA and examined for p44/42 MAPK, p38 MAPK and AKT activation. We found that exposure of PECs to albumin caused significant and sustained increase in phosphorylation of p44/42 MAPK but not p38 MAPK (Fig 7). Furthermore, incubation of PECs with U0126, a selective inhibitor of p44/42 MAPK, resulted in a significant reduction of MMP-9 activity and protein in culture supernatants following RSA (0.25 mg/ml) treatment (Fig 8). AKT phosphorylation was low in both untreated and RSA-treated glomerular PECs.

### Effect of High Glucose and TGF-β1 on MMP-9 in Primary PECs

**Fig 9:**
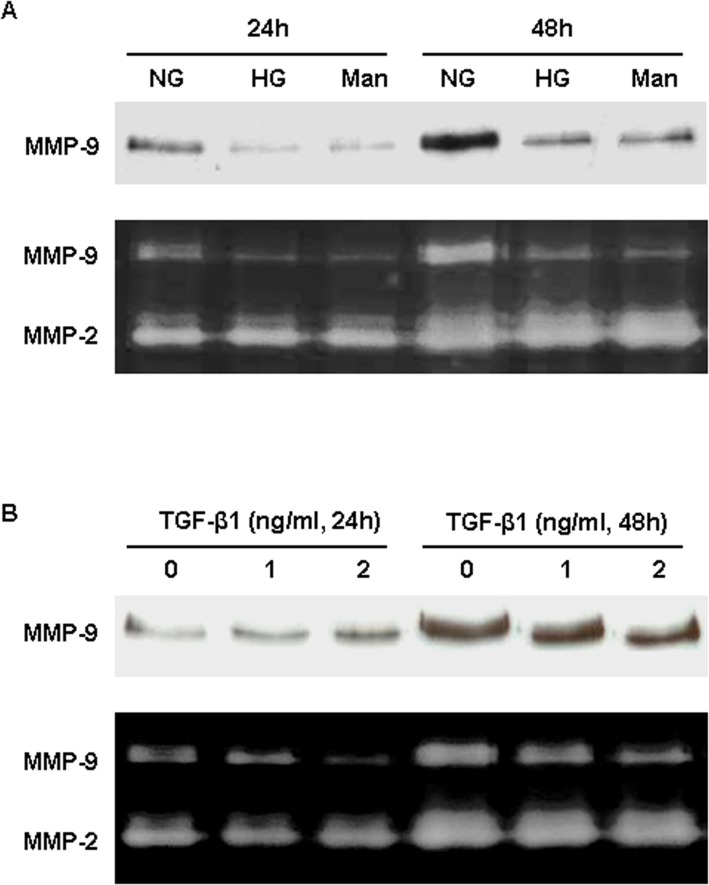
Effects of high glucose and TGF-β1 on MMP-9 secretion by glomerular PECs. (A) MMP-9 protein and activity in culture supernatants of PECs treated with normal glucose (NG), high glucose (HG) or mannitol (Man) for 24 or 48 hrs. (B) MMP-9 protein and activity in culture supernatants of PECs treated with recombinant TGF-β1 (1 or 2 ng/ml) for 24 or 48 hrs.

High-glucose condition has been shown to modify MMP production by glomerular podocytes [28] and mesangial cells [29]. Here, we further evaluated the effect of high-glucose on gelatinases in primary PECs. A reduction in MMP-9 protein and activity in culture supernatants at 24 and 48 hr was detected when the primary PECs were incubated with 30 mM glucose compared to 5 mM glucose (Fig 9A). This decrease was also seen in the osmotic control cells in which mannitol was added to keep the same osmolarity as that under condition of high concentration of glucose (Fig 9A). MMP-2 protein and activity were not significantly modified by high-glucose or mannitol treatment.

Previous studies also show that TGF-β1 is capable of inducing MMP-9 expression and activity in podocyte [30] and tubule [11, 31] cell lines. To study the response of glomerular PECs to TGF-β1, we measured MMP-9 protein and activity in culture supernatants. We found that TGF-β1 (1–2 ng/ml) decreased MMP-9 protein and activity in culture supernatants of PECs, whereas MMP-2 activity was barely affected (Fig 9B).

## Discussion

Dysregulation of glomerular MMP-9 expression and/or activity has been demonstrated in both animal models [24, 32] and patients [33–34] with proteinuric renal diseases. In this study, we further report a focal increase in glomerular gelatinase activity and MMP-9 expression in parietal epithelium in association with a significant loss of adjacent podocytes in the damaged glomeruli of diabetic animals. A stimulatory effect of albumin on MMP-9 expression and secretion was detected in primary cultured rat glomerular PECs. These findings identify albumin as a signaling molecule that can stimulate MMP-9 production by activated glomerular parietal cells, which may play an important role in PEC migration and podocyte dysfunction during the development and progression of diabetic nephropathy.

The gelatinases, MMP-2 and MMP-9, are involved in the degradation of glomerular ECM components, particularly type IV collagen. In normal kidneys, MMP-9 expression has been observed in both mesangial cells [24] and glomerular visceral epithelial cells (podocytes) [15, 35]. Using immunohistochemistry and double-label immunofluorescence microscopy, Kuroda et al. showed that MMP-9 was localized in the mesangial cells in glomeruli of normal rat kidneys [24]. In line with these findings, using in situ zymography we detected the presence and localization of gelatinase activity in the mesangial area of normal rat glomeruli. Dual labeling for MMP-9 and desmin confirmed expression of MMP-9 by desmin-positive mesangial cells in normal glomeruli. There was no significant colocalization between MMP-9 and podocyte marker proteins. Our results support that mesangial cells are a source of MMP-9 in the glomerular tuft in normal rat kidneys. One interesting observation in the present study is that the gelatinolytic activity and expression of MMP-9 were focally increased in the damaged glomeruli in Zucker diabetic rats. Moreover, increased MMP-9 signal was often detected in the area with low desmin staining in the damaged glomeruli of diabetic rats. A colocalization of MMP-9 and claudin-1 suggests that increased MMP-9 signal was present in claudin-1-positive parietal epithelium. Cell lineage tracing experiments are warranted on unequivocally determining whether MMP-9 expressing cells within sclerotic lesions of the affected glomeruli originate from PECs.

Glomerular podocytes and PECs derive from a common ancestral mesenchymal cell and begin to acquire individual cell characteristics by the S-shaped body phase of glomerulogenesis [4]. Normally, PECs are inconspicuous flat cells with morphologically tight intercellular junctions and primary cilia. In glomerular diseases, PECs may serve a critical reparative role due to its proliferative capacity, or under different circumstances the response by PECs may lead to further glomerular damage [6]. We have previously demonstrated that Zucker diabetic fatty rats have increased PEC proliferation and migration in association with podocytopenia in the damaged glomeruli [9]. Here, we further report that the activated PECs exhibited high MMP-9 signal and gelatinolytic activity in the glomeruli of diabetic rats. Since MMP-9 has been shown to play an important role in cell migration and tissue remodeling, we speculate that striking MMP-9 induction in activated PECs would accelerate the progression of diabetic glomerulopathy by promoting PEC migration and podocyte dysfunction.

Recent cell lineage tracing studies performed by Smeets et al. have clearly demonstrated a role of PECs in the pathogenesis of glomerular sclerotic lesions [2, 7]. These reports indicate that activated PECs could migrate onto the glomerular tuft, replace the podocytes, and deposit BC-type matrix in different models of FSGS [2, 7]. These observations support that the activation and migration/invasion of PECs might represent a common pathologic phenomenon in very different glomerular diseases that eventually lead to glomerular scarring. However, the exact mechanisms underlying PEC migration and podocyte dysregulation have not been fully understood. It is well known that MMP-9 plays an important role in the invasion of cancer cells including renal carcinoma [36]. Therefore, an identification of increased MMP-9 in activated PECs may provide an explanation of enhanced PEC proliferation and migration in diabetic kidney disease. Activation of PECs in association with de novo expression of CD44 has been shown in three distinct models of FSGS [2, 7]. Interestingly, docking of MMP-9 at the cell surface by CD44 was reported to promote MMP-9-mediated cell migration [37–39]. Based on these findings, we further speculate that MMP-9 may promote activated parietal cell migration onto the glomerular tuft via its interaction with CD44.

In the present study, we also found that the appearance of MMP-9 positive PECs on the glomerular tuft was frequently associated with the loss of podocyte marker proteins in affected glomeruli in advanced stages of diabetic nephropathy. A decrease in podocyte markers could be due to podocyte dedifferentiation or detachment from the glomerular basement membrane (GBM). Increased urinary excretion of podocyte marker proteins in the diabetic rats highlights that detachment of podocytes from the GBM rather than dedifferentiation may contribute to the podocyte loss. This is also supported by the previous finding that podocyte detachment was increased in correlation with GBM thickness, albuminuria and fractional mesangial area in patients with type 2 diabetes [40]. The associated loss of podocytes/glomerular filter integrity in the damaged glomeruli may reflect proteolytic degradation of the GBM by locally increased gelatinases produced by invaded PECs in our diabetic animal model. Therefore, it is likely that podocytes can be lost secondarily because of the invasion of PECs, which express and secret high levels of MMP-9 alone or in combination with other proteases in advanced diabetic kidney disease.

So, next question is what stimulates MMP-9 expression and secretion by activated PECs in disease condition. Increased urinary albumin excretion is a hallmark of glomerular disease including diabetic nephropathy. Albumin has been shown to upregulate MMP-9 expression and secretion in cultured podocytes [41]. Therefore, we further hypothesized that PECs are sensitive to an elevation of albumin in the Bowman’s space and can produce MMP-9 upon activation. To test this hypothesis, we determined the effect of albumin overload on MMP-9 expression and secretion by primary cultured rat glomerular PECs expressing high-level of claudin-1 protein. As expected, albumin stimulated MMP-9 production as evidenced by an elevation of MMP-9 protein and activity in culture supernatants of primary glomerular PECs. In contrast, albumin had no effect on MMP-2 expression. Our mechanistic studies further demonstrate the involvement of p44/42 MAPK activation in albumin-mediated MMP-9 induction. The phosphorylation of ERK1/2 but not p38 MAPK was significantly increased when the primary PECs were incubated with albumin. Moreover, albumin-induced enzymatic activity and secretion of MMP-9 were suppressed markedly in the presence of U0126, a selective p44/42 inhibitor. Although our in vitro findings support that albumin may induce MMP-9 activity and secretion by PECs through the ERK signaling pathway, further in vivo studies are required to validate the stimulatory role of albumin overload on parietal MMP-9 in diabetic glomerulopathy.

Because extracellular protease activity requires efficient release of these proteases to the cellular surface, we investigated the expression and intracellular localization of MMP-9 in cultured glomerular PECs. Immunolabeling of PECs with antibody specific for MMP-9 led to the identification of MMP-9 in small cytoplasmic vesicles. In combination with α-tubulin-specific antibody, MMP-9-positive vesicles were found to be mainly aligned along the microtubular network. Normally, the PECs form an impermeable barrier through the formation of tight junctions, preventing glomerular ultrafiltrate constituents from exiting Bowman’s space into the periglomerular interstitial space. Increased MMP-9 protein would impair the integrity of cell-cell contact by disrupting zonula occludens-1 (ZO-1), a key component of tight junctions, which has been identified as an in vivo MMP-9 substrate [42]. Additionally, MMP-9 is known to be capable of cleaving osteopontin (OPN), a macrophage chemoattractant. In fact, parietal expression of OPN was upregulated in activated PECs in the diabetic rats (data not shown). Together, increased local activity of MMP-9 could exacerbate glomerular injury by driving the turnover of extracellular matrix proteins and interfering with cell-cell interactions and signaling molecules.

Induction of MMP-9 after high glucose [28] or TGF-β1 [30] stimulation has been demonstrated in podocytes. In the current study, incubation of PECs with high glucose resulted in a decrease in MMP-9 production. This reduction was mimicked by mannitol, given to deliver the same osmolarity to the cells. Recently, one group reported that high glucose (25 mM) reduces the activities of MMP-2 and MMP-9 in a cell line of rat mesangial cells [29], but it is unclear whether this result was due to a metabolic or an osmotic effect. Our result supports that the inhibitory effect of high glucose on glomerular PEC MMP-9 activation is largely a consequence of increased osmolality, although the molecular mechanism by which osmolar stress leads to an inhibition of MMP-9 activation remains to be elucidated. By contrast, neither glucose nor mannitol had effect on MMP-2 expression and activity in PECs. A moderate suppression of MMP-9 secretion was also observed when the PECs were exposed to TGF-β1 (1–2 ng/ml) for 24 or 48 hrs. Our results suggest that glucose or TGF-β1 per se may play a minor role in local activity of MMP-9 in the diabetic glomeruli.

In summary, our data show focally increased glomerular gelatinolytic activity and MMP-9 expression in activated PECs in the diabetic kidneys. Notably, an upregulation of parietal MMP-9 was associated with an increase in urinary excretion of podocyte marker proteins and a consequent reduction of podocytes in the damaged glomeruli. Albumin overload stimulated MMP-9 release from cultured PECs through an activation of p44/42 MAPK signaling pathway. Our results support that inhibition of PEC activation and MMP-9 production may represent a major opportunity for the prevention and treatment of proteinuric glomerular diseases.
